# A Comparison of Whole-Brain Dose During Stereotactic Radiosurgery for Multiple Metastases Across Technology Platforms

**DOI:** 10.7759/cureus.92442

**Published:** 2025-09-16

**Authors:** Neelan J Marianayagam, Ziyi Wang, Alonso N Gutierrez, D Jay Wieczorek, Ian Paddick, Yusuke S Hori, David J Park, Steven D Chang, Stephanie Key, Georg A Weidlich, John Adler

**Affiliations:** 1 Neurosurgery, Stanford University, Stanford, USA; 2 Radiation Oncology, Stanford Health Care Cancer Center, Palo Alto, USA; 3 Radiation Oncology, Florida International University, Herbert Wertheim College of Medicine, Miami, USA; 4 Radiation Oncology, Miami Cancer Institute, Miami, USA; 5 Medical Physics, Medical Physics Limited, London, GBR; 6 Neurosurgery, Stanford University School of Medicine, Stanford, USA; 7 Sales and Marketing, ZAP Surgical Systems, Inc., San Carlos, USA; 8 Medical Physics, ZAP Surgical Systems, Inc., San Carlos, USA; 9 Radiation Oncology, ZAP Surgical Systems, Inc., San Carlos, USA

**Keywords:** gamma knife (gk) radiosurgery, gyroscopic radiosurgery, linac-based radiosurgery, stereotactic radiosurgery, treatment of brain metastases, whole brain radiation dose

## Abstract

Stereotactic radiosurgery (SRS) is now the gold standard radiation technique for the treatment of multiple brain metastases. By virtue of its targeting accuracy, SRS, relative to whole-brain radiotherapy (WBRT), maximizes both the likelihood of tumor ablation while minimizing irradiation (injury) of the uninvolved brain. The extent to which the latter objective is accomplished varies among radiosurgery platforms. In this study, we used a standardized imaging dataset taken from a patient with 10 random brain metastases to calculate the radiation dose delivered to the uninvolved normal brain across a range of modern SRS delivery platforms. This analysis reveals that irradiation of the uninvolved brain is considerably less on dedicated cranial SRS devices compared to multi-purpose C-arm and full-body robotic systems. Given the growing recognition of lower-dose radiation’s deleterious effects, these findings may have relevance to patient and technology selection.

## Introduction

By virtue of minimizing neurocognitive damage, “highly focal” stereotactic radiosurgery (SRS) is now deemed superior to whole-brain radiotherapy (WBRT) in the treatment of multiple brain metastases [1-4]. Although the exact radiobiologic mechanism remains cryptic, “low-dose” radiation exposure appears to damage cerebrovasculature, increase neuroinflammation, and impair neurogenesis, which collectively alter normal brain functions [2]. The greater spatial precision and accuracy of SRS appear to lessen these highly deleterious side effects. However, the extent of uninvolved brain irradiation varies among radiation delivery platforms, especially in patients with multiple metastases. Despite the current ubiquity of brain SRS for brain metastases, little has been published quantifying the dosimetric differences among different radiosurgical devices. In this study, we seek to make a rigorous comparison among a wide range of SRS technologies that are commonly used to treat multiple brain metastases.

The most widely used metrics for establishing the relative quality of radiosurgical treatment are dose conformity (CI) and gradient (GI) indices [5,6]. However, as a secondary and absolute measure, V12 Gy (the volume of brain receiving a dose of greater than 12 Gy) is also deemed an important predictor of radiation-induced brain injury (RIBI), which is clinically characterized by brain enhancement and edema on contrast magnetic resonance imaging (MRI) brain, and often accompanied by conclusive brain necrosis on histological examination [7]. Given that such damage has the obvious potential to cause cognitive decline and executive dysfunction, it is featured prominently in modern clinical trial designs and guidelines [7,8]. Nevertheless, even less severe forms of radiation injury have been shown to correlate with cognitive decline and impaired executive function, despite the absence of radiographic changes - with fractionated WBRT serving as a notable example. Furthermore, more recent animal research unambiguously demonstrates that lower radiation doses, e.g., as low as the V5 Gy, represent an even lower “non-lesional” threshold for altering local neural circuit activity and damaging brain function. These recent findings elevate the clinical importance of low-dose brain radiation far beyond anything that was previously appreciated [9]. As brain metastasis patients continue to live longer, and thus the incidence of new and recurrent metastatic brain disease grows, this phenomenon is of particular concern. In light of these new findings, lower doses of radiation “spillage” now merit special consideration when designing treatment plans for multi-metastasis patients.

## Technical report

Materials and methods

A single de-identified contrast computerized tomography (CT) and contrast MRI (T1 MR slice thickness included 1.25 mm with 512x512 image resolution) obtained in a single patient with 10 widely dispersed brain metastases was obtained from Stanford University (Stanford, CA) and used throughout this study. Ten target volumes were identified as the gross tumor volumes (GTVs), ranging in maximum dimension from 2.1 mm to 5.0 mm, with a median of 3.3 mm, and in volume from 0.01 cm^3^ to 0.06 cm^3^, with a median of 0.02 cm^3^. This solitary dataset was then provided to a series of expert users across multiple SRS platforms from multiple institutions, each of whom generated clinically deliverable and relevant treatment plans befitting routine clinical practice and within reasonable and standard treatment time slots for their respective clinical practice. The resulting plans were analyzed and compared. Multi-purpose radiation delivery systems analyzed as part of this study include the following: (1) Varian TrueBeam multi-purpose C-arm delivery platform (Palo Alto, CA) - standard radiotherapy configuration, (2) Varian TrueBeam Edge multi-purpose C-arm delivery platform (Palo Alto, CA) - stereotactic radiosurgery configuration, and (3) Accuray CyberKnife M6 full-body robotic radiosurgery platform (Sunnyvale, CA). The dedicated cranial radiosurgery delivery systems analyzed as part of this study include the following: (1) Elekta Gamma Knife - Esprit Cobalt-60 radiosurgery platform (Stockholm, Sweden), (2) Elekta Gamma Knife - Icon Cobalt-60 radiosurgery platform (Stockholm, Sweden), and (3) ZAP Surgical - ZAP-X gyroscopic radiosurgery platform (San Carlos, CA). The following section describes the methodology used to generate specific treatment plans for each of the respective delivery systems.

Varian TrueBeam multi-purpose C-arm delivery platform - standard radiotherapy configuration

**Figure 1:**
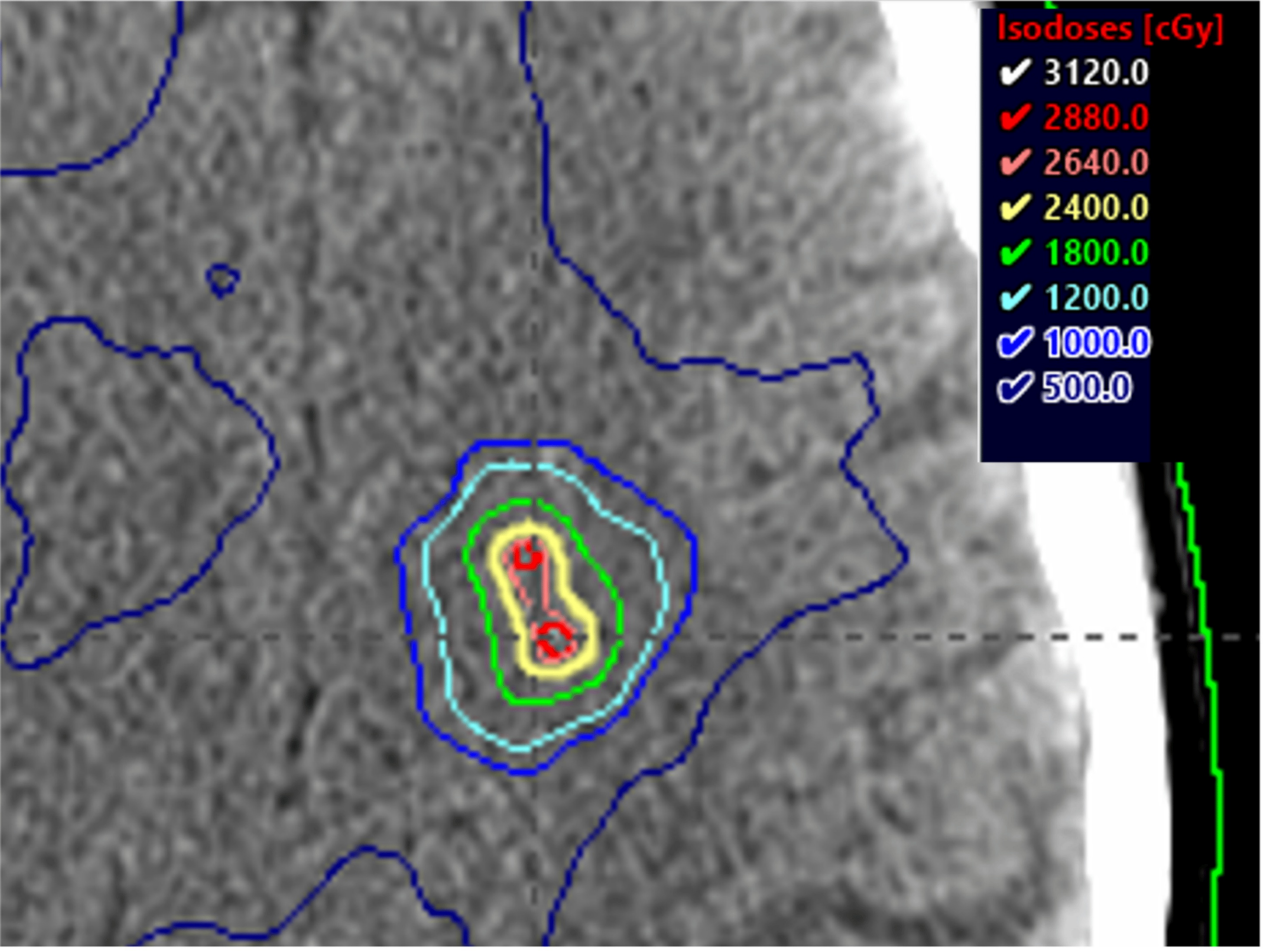
Varian TrueBeam single isocenter treatment plan showing two adjacent metastases of the 10 metastases standardized patient. TrueBeam (Palo Alto, CA: Varian).

For the Varian TrueBeam LINAC (Palo Alto, CA) (Stanford LA20, Series #6221) with a Millennium 120 MLC (leaf width 5 mm), a single isocenter was selected near the geometric center of 10 target volumes. Gross tumor volumes (GTVs) were expanded volumetrically using a setup margin of 1 mm to create a corresponding planning target volume (PTV). One coplanar partial arc and three non-coplanar partial arcs (table angles: 15°, 30°, and 45°) were selected to encompass all target volumes. Varian Eclipse v15.6 (Palo Alto, CA) (PO_15605) volumetric modulated arc therapy (VMAT) optimization with an anisotropic analytical algorithm (AAA) calculation model was used with a 1.25 mm dose grid. Three VMAT iterations were performed to achieve a plan with 99% prescription dose coverage of all target volumes, while minimizing dose to the optic pathways, brainstem, and uninvolved brain. The treatment plan with overlaid isodose lines is shown in Figure 1.

Varian TrueBeam Edge multi-purpose C-arm delivery platform - stereotactic radiosurgery configuration

The 10 targets’ gross tumor volumes (GTV) were imported into the Varian Eclipse treatment planning system (version 15.610), and the GTVs were expanded volumetrically using a setup margin of 1 mm to create a corresponding planning target volume (PTV). Using a TrueBeam Edge with the high definition (HD) multi-leaf collimator (MLC) (2.5 mm inner leaf width), two plans were created to treat each of the PTVs to at least 99.5% prescription dose coverage while minimizing dose to the normal brain. Plans were optimized using Eclipse PO_15.6.06 VMAT optimizer and calculated using ACUROS_15.6.06 with a 1 mm dose grid.

**Figure 2:**
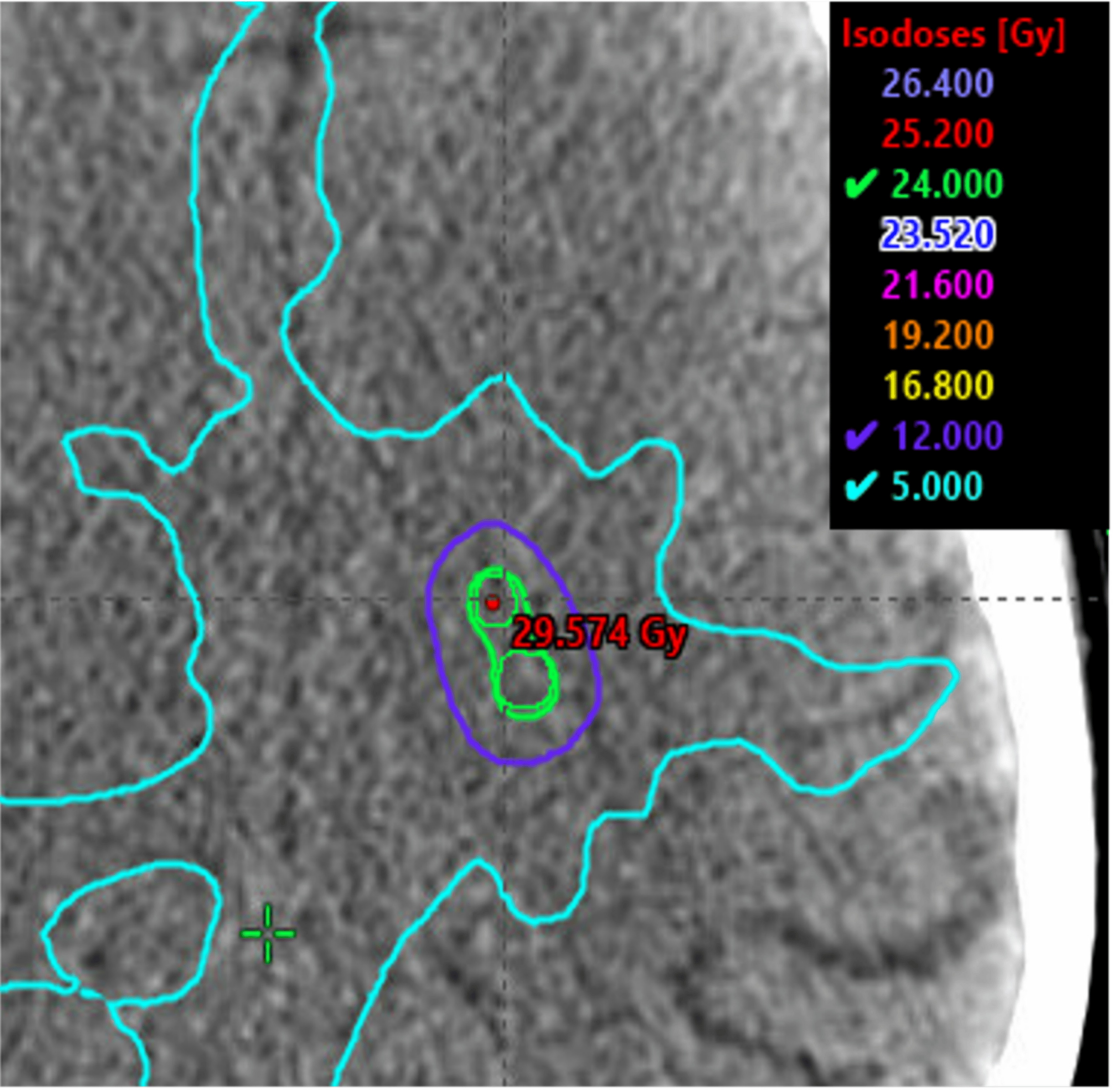
Varian TrueBeam Edge single isocenter treatment plan showing two adjacent metastases of the 10 metastases standardized patient. TrueBeam Edge (Palo Alto, CA: Varian).

The single isocenter plan was created using one coplanar and two non-coplanar arcs (table angles: 45° and 315°). The mean prescription isodose lines for all targets were 76% (69-83%). The treatment plan with overlaid isodose lines is shown in Figure 2.

**Figure 3:**
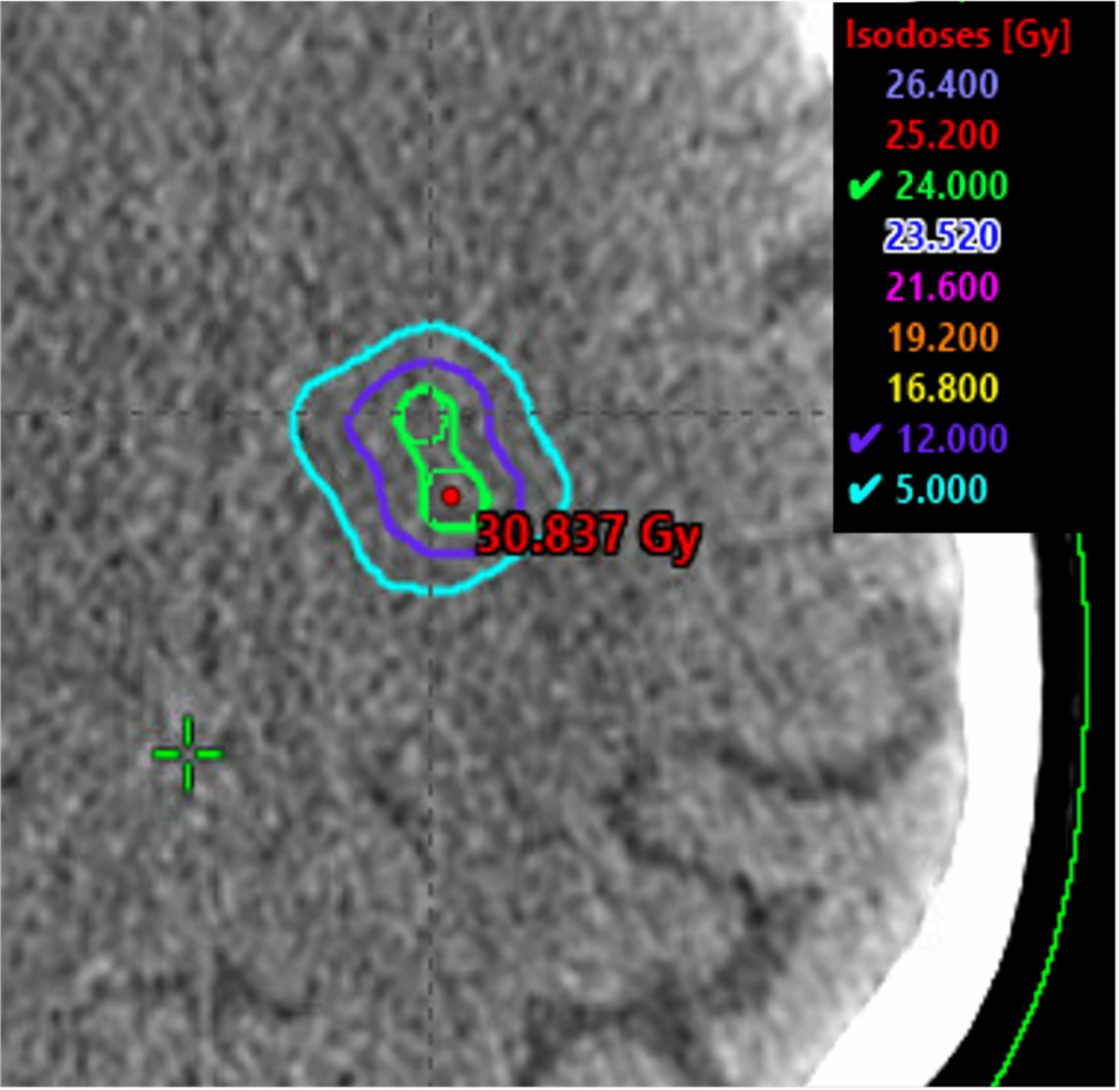
Varian TrueBeam Edge three isocenter treatment plan showing two adjacent metastases of the 10 metastases standardized patient. TrueBeam Edge (Palo Alto, CA: Varian).

The three-isocenter plan was a composite of three individual plans, each with a distinct isocenter targeting a separate set of PTVs. Each individual plan consists of three coplanar and/or non-coplanar beams. Several iterations were performed until the composite plan achieved the desired target coverage for each PTV, optimized prescription dose conformity and dose fall-off, and minimized normal brain dose. The mean prescription isodose lines for all targets were 75% (69-78%). The treatment plan with overlaid isodose lines is shown in Figure 3.

Accuray CyberKnife M6 full-body robotic radiosurgery platform

**Figure 4:**
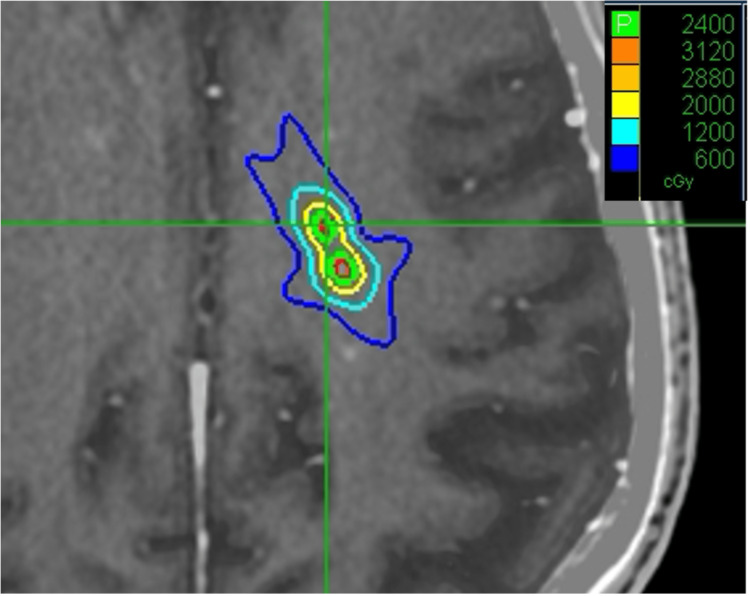
Accuray CyberKnife M6 treatment plan showing two adjacent metastases of the 10 metastases standardized patient. CyberKnife M6 (Sunnyvale, CA: Accuray Incorporated).

For CyberKnife M6 (Sunnyvale, CA: Accuray Incorporated), the accuracy and precision treatment planning system 3.5.0.1 (3) was used to generate the data. VOLO (Sunnyvale, CA: Accuray Incorporated) optimization with a ray-tracing calculation model was used with a 1.25 mm dose calculation grid. Fifty-nine non-isocentric nodes of 83 beams were selected by the VOLO optimizer and a fixed 7.5 mm cone was used for each of the 10 target volumes. Seventy optimization iterations were performed to achieve 98% prescription dose coverage of target volumes, while minimizing dose to the optical structures, brainstem, and uninvolved brain to as low as possible. The prescription isodose line was 76.4%. The treatment plan with overlaid isodose lines is shown in Figure 4.

Elekta Gamma Knife Esprit Cobalt-60 radiosurgery platform (dedicated cranial SRS)

**Figure 5:**
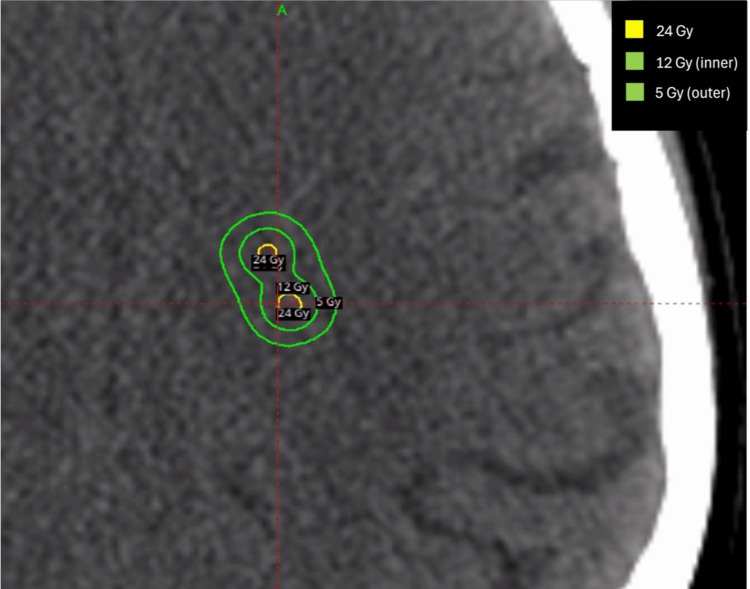
Elekta Gamma Knife Esprit treatment plan showing two adjacent metastases of the 10 metastases standardized patient. Elekta Gamma Knife Esprit (Stockholm, Sweden: Elekta).

The 10 GTV targets were imported into the Leksell GammaPlan (LGP) version 11.4.2 (Stockholm, Sweden: Elekta). The Lightning dose optimizer (Stockholm, Sweden: Elekta) was used to generate an initial plan with the default 0.5/0.5 (low-dose/beam-on time) optimization setting parameters and with the coverage option enabled. The initial plan was then assessed visually for coverage, conformity, and dose fall-off, and was manually adjusted to achieve at least 99.5% prescription dose coverage to each GTV, as well as an optimal Paddick Conformity Index (PCI). The mean prescription isodose line for all targets was 81% (61-93%) for the plan. The treatment plan with overlaid isodose lines is shown in Figure 5.

Elekta Gamma Knife Icon Cobalt-60 radiosurgery platform (dedicated cranial SRS)

**Figure 6:**
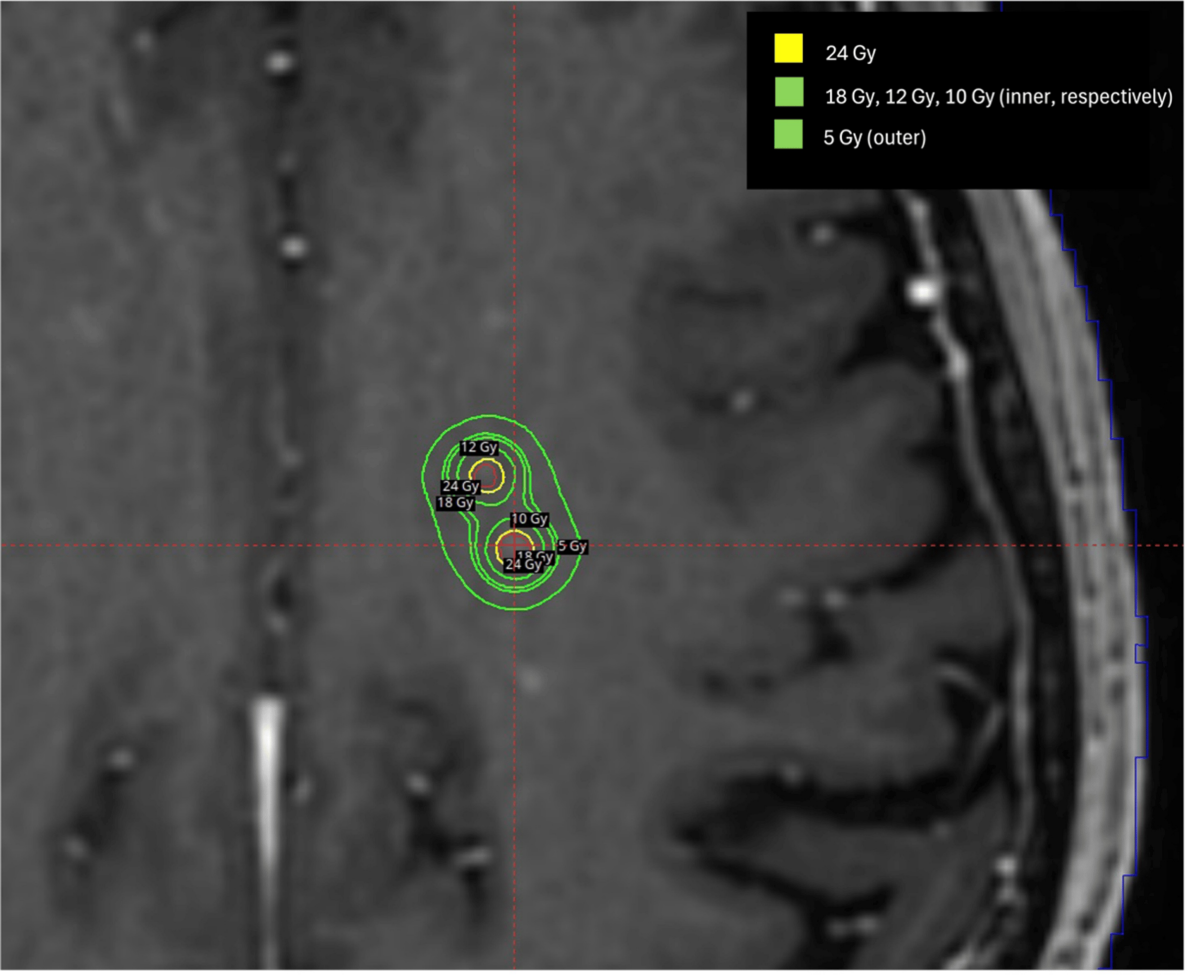
Elekta Gamma Knife Icon treatment plan showing two adjacent metastases of the 10 metastases standardized patient. Elekta Gamma Knife Icon (Stockholm, Sweden: Elekta).

Leksell GammaPlan version 11.4 (Stockholm, Sweden: Elekta) was used with a forward planning approach. A single isocenter was selected for each GTV with no additional margin added. The diameter of the collimator for each target was selected to most closely match the mean target diameter. A tissue maximum ratio (TMR) based dose calculation was performed with a 0.5 mm dose grid. The prescription isodose was adjusted to maximize selectivity while maintaining 99-100% coverage of the target with the prescription dose. This ranged between the 57% and 93% isodose depending on the collimators used and the volume of the target. Treatment plan with isodose lines shown in Figure 6.

ZAP Surgical ZAP-X gyroscopic radiosurgery platform (dedicated cranial SRS)

**Figure 7:**
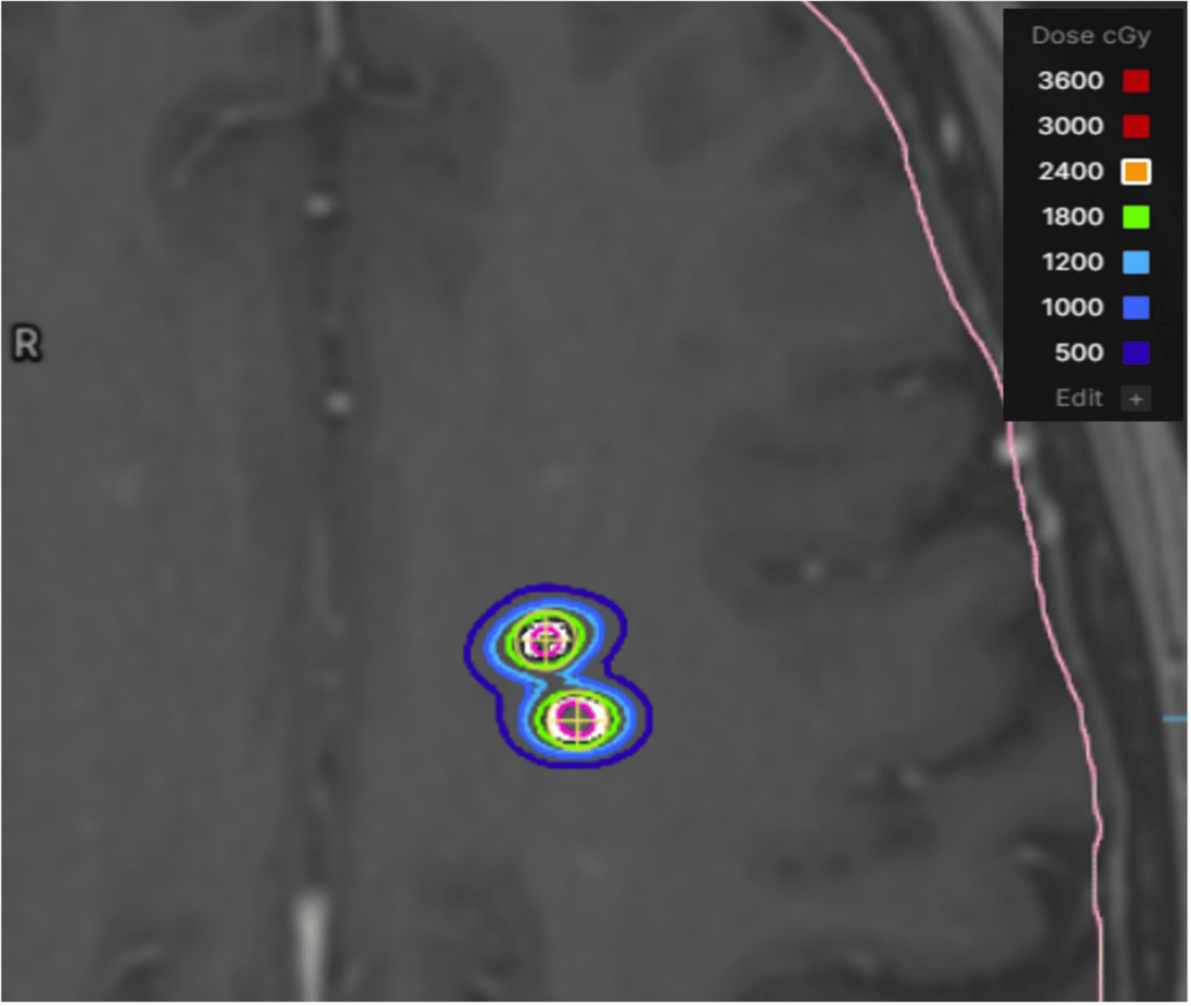
ZAP Surgical ZAP-X treatment plan showing two adjacent metastases of the 10 metastases standardized patient. ZAP-X (San Carlos, CA: ZAP Surgical Systems, Inc.).

Using the ZAP-AXON planning system (San Carlos, CA: ZAP Surgical Systems, Inc.) (pending US FDA 510(k) clearance), a single isocenter was selected for each GTV target, and the diameter of the collimator was selected to most closely match the maximum target diameter. No additional margin was added to the GTV targets. Dose calculation in ZAP-AXON is based on the ray-tracing algorithm with a 1 mm dose grid. Inverse optimization was performed to maximize target coverage and minimize dose to the uninvolved brain. Prescription isodose lines were selected to achieve 100% coverage of each target; individual isodose line prescriptions for each GTV ranged from 51-89%. Treatment plan with isodose lines shown in Figure 7.

Results

In Table 1, we show seven treatment planning and dosimetric parameters across the six SRS systems utilized in this study of a single standardized patient with 10 brain metastases. Among the treatment plans, three utilize multi-purpose, full-body LINAC systems, comprising two C-arm-based designs and one robotic, while the other three are dedicated to cranial radiosurgery applications. The two Cobalt-60 platforms represent different models of Gamma Knife systems deployed at separate institutions. It should be noted that the C-arm LINAC platforms utilize a 1 mm margin expansion as a standard practice when treating brain metastases with a single isocenter using multiple VMAT arcs. This same expansion is not used with dedicated cranial radiosurgery platforms or the CyberKnife, with the former notably utilizing multiple individual isocenter treatment plans. Importantly, treatment planning for robotic (CyberKnife) radiosurgery is non-isocentric.

**Table 1:** Treatment planning and dosimetric parameter comparison across SRS delivery systems for a single 10 metastases case. SRS: stereotactic radiosurgery; GTV: gross tumor volumes; PTV: planning target volume; tx: treatment; est: estimated

Variables	Multi-purpose C-arm and full-body robotic delivery platforms				Dedicated cranial SRS platforms		
	Varian TrueBeam	Varian TrueBeam Edge	Varian TrueBeam Edge	Accuracy CyberKnife M6	Elekta Gamma Knife Esprit	Elekta Gamma Knife Icon	ZAP Surgical ZAP-X
Prescription dose/coverage	24 Gy @ 99%	24 Gy @ 99.58%	24 Gy @ 99.48%	24 Gy @ 98%	24 Gy @ >99.5%	24 Gy @ 99-100%	24 Gy @ 100%
GTV-PTV margin expansion (based on common standard of practice)	1 mm	1 mm	1 mm	0 mm	0 mm	0 mm	0 mm
Isocenter(s)	1	1	3	Non-isocentric. CK nodes=59	11	10	10
Beam array	1 full coplanar axial arc, 1 full non-coplanar 15° arc, and 2 partial non-coplanar arcs (3 total couch kicks)	1 full coplanar axial arc and 2 non-coplanar partial arcs (2 total couch kicks)	2 full coplanar arcs and 7 non-coplanar partial arcs (7 total couch kicks)	83 non-coplanar beams	192 fixed, non-coplanar beams per isocenter	192 fixed, non-coplanar beams per isocenter	Total of 802 unique non-coplanar beams (average 80.2 beams per isocenter)
Beam energy	6 MV linear accelerator (flattening filter free {FFF})	6 MV linear accelerator (flattening filter free {FFF})	6 MV linear accelerator (flattening filter free {FFF})	6 MV linear accelerator (flattening filter free {FFF})	1.25 MeV Co-60 sources	1.25 MeV Co-60 sources	3 MV linear accelerator (flattening filter free {FFF})
Source-axis distance (SAD)	100 cm	100 cm	100 cm	80 cm	~40 cm	~40 cm	45 cm
Collimation	120 leaf MLC, 5 mm inner leaf width leakage spec: <3.0%	120 leaf MLC, 2.5 mm inner leaf width leakage spec: <2.5%	120 leaf MLC, 2.5 mm inner leaf width leakage spec: <2.5%	Fixed 7.5 mm cone leakage spec: <0.2%	4 mm leakage spec: <0.001% (at 70 cm)"	4 mm leakage spec: <0.001% (at 70 cm)	4 mm leakage spec: <0.001% (at 1 m)
Target max dose	32.36 Gy	34.98 Gy	34.86 Gy	31.4 Gy	39.30 Gy	42.10 Gy	44.53 Gy
Whole brain V12	22.90 cc	10.70 cc	8.79 cc	7.29 cc	1.63 cc	1.67 cc	1.74 cc
Whole brain V10	38.20 cc	16.66 cc	12.75 cc	9.6 cc	2.15 cc	2.20 cc	2.35 cc
Whole brain V5	332.10 cc	122.70 cc	64.61 cc	35.35 cc	6.22 cc	6.49 cc	7.29 cc
Tx time (door-to-door)	25 min (est)	30 min (est)	70 min (est)	56 min (est)	149 min (est)	144 min (est)	115 min (est)

**Figure 8:**
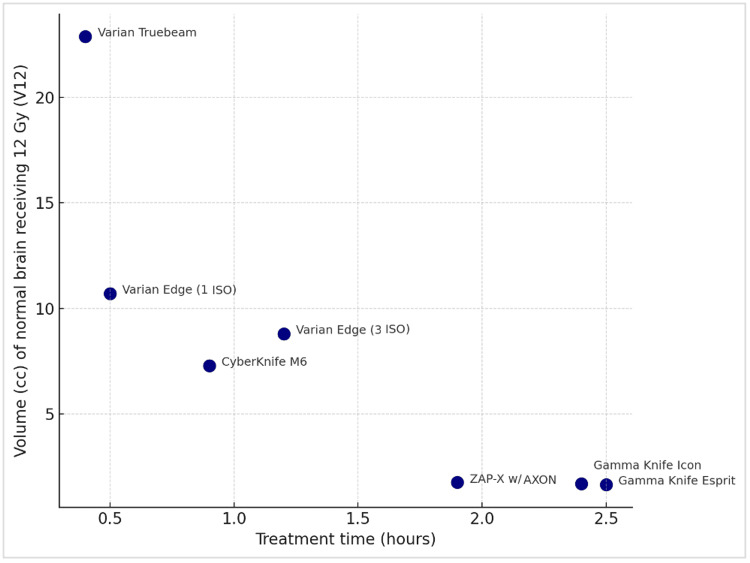
Volume (cc) of normal brain receiving 12 Gy (V12 Gy) versus treatment time. Elekta Gamma Knife treatment times were adjusted to a reference dose rate of 2.52 Gy/min, which corresponds to sources that are approximately halfway through their five-year lifespan (i.e., 2.5 years).

**Figure 9:**
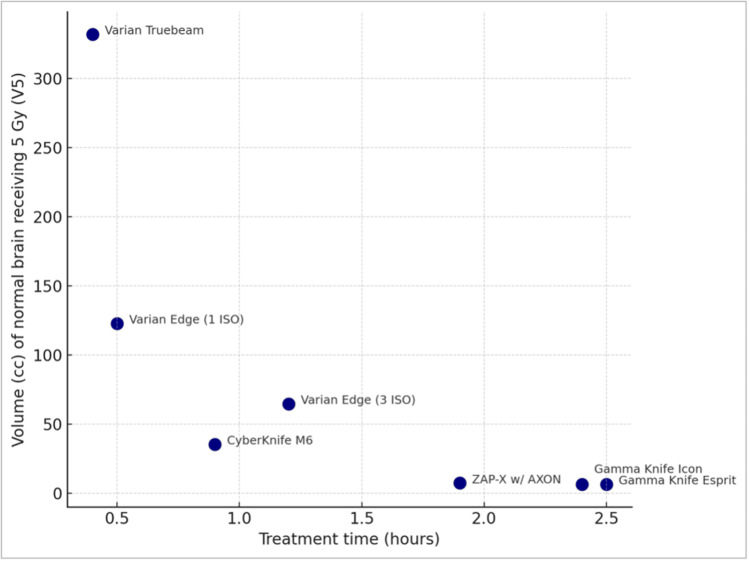
Volume (cc) of normal brain receiving 5 Gy (V5 Gy) versus treatment time. Elekta Gamma Knife treatment times were adjusted to a reference dose rate of 2.52 Gy/min, which corresponds to sources that are approximately halfway through their five-year lifespan (i.e., 2.5 years).

Table 1 depicts the uninvolved brain volume receiving 12 Gy, 10 Gy, and 5 Gy (V12 Gy, V10 Gy, and V5 Gy, respectively). These values vary substantially across different SRS platforms, with V12Gy ranging from 22.90 to 1.63 cc, V10 Gy from 38.20 to 2.15 cc, and V5 Gy from 332.10 to 6.22 cc. Figure 8 is a scatter plot showing volume (cc) of normal brain receiving 12 Gy (V12 Gy) versus treatment time. Figure 9 is a scatter plot that visually depicts the volume (cc) of the normal brain receiving 5 Gy (V5 Gy) versus treatment time. Overall, the whole brain radiation exposure is best minimized in platforms dedicated to cranial radiosurgery, with the Gamma Knife units slightly superior to those of ZAP-X. The technical reasons for this wide performance disparity (e.g., over 50-fold variation in V5 Gy) as well as the possible clinical consequences will be presented in the discussion to follow.

## Discussion

In this study, we demonstrate, in a setting of treating multiple brain metastases, a significant variation in the amount of lower-dose radiation (V12 Gy, V10 Gy, and V5 Gy), encompassing the normal brain across a wide spectrum of commercially available radiosurgical platforms. What is most striking is the significantly smaller low-dose volumes of dedicated cranial SRS platforms relative to multi-purpose C-arm and full-body robotic systems, with a 50-fold variation being observed in the most extreme (V5 Gy) situation.

There are several reasons for the larger whole brain exposure observed on the multi-purpose C-arm and full-body robotic systems, including (i) higher collimator leakage is intrinsic to multi-purpose C-arm Linacs and full-body robotic systems when compared to the dedicated cranial radiosurgery systems (<3.0% for TrueBeam, <2.5% for TrueBeam Edge, and <0.2% for CyberKnife, versus <0.001% for Gamma Knife and ZAP-X); (ii) larger source axis distance (SAD) in the multi-purpose C-arm and full-body robotic systems results in larger radiation penumbra (100 cm and 80 cm, respectively, versus 40-45 cm); (iii) higher scatter beam energy is observed in the higher-energy multi-purpose C-arm and full-body robotic systems (6 MV versus 3 MV). It has been shown that the beam quality factor of cobalt-60 (1.0) is similar to the beam quality factor of ZAP-X (0.9964) [10]. Consequently, the beam quality and radiation scatter characteristics of both platforms are comparable; and (iv) when treated in single isocenter volumetric modulated arc therapy (VMAT) mode, multi-purpose C-arm systems require wide open primary jaws, resulting in a higher dose to the unaffected brain. Since beam collimation for each metastasis is exclusively accomplished by the multi-leaf collimator (MLC), the resulting higher radiation interleaf leakage radiation causes more dose to be delivered outside of the targeted volume.

The reader will note that door-to-door treatment time is considerably shorter on multi-purpose C-arm systems. There appears to be a clear trade-off between speed (e.g., single isocenter SRS) and quality (e.g., multiple isocenter SRS). Although single-isocenter multi-purpose C-arm radiosurgery is shown to be both safe and effective in the treatment of multiple brain metastases, the need to accommodate less accurate intrafraction targeting also contributes to more radiation spillage onto normal brain [11,12].

This study has important limitations. First, it is based on a single patient dataset, which, while anatomically representative, does not capture the full variability of target size, depth, or spatial distribution seen in clinical practice. Second, all plans were generated by expert users on their own platforms, which reflects real-world expertise but may bias performance toward familiar systems. Third, margin practices differed across platforms; although this reflects standard workflows, it may influence low-dose exposure. Fourth, the study is purely dosimetric and does not evaluate whether lower V5-V12 exposures translate into improved preservation of neurocognitive function. Finally, technology continues to evolve, including new collimators, algorithms, and source configurations, so results reflect the specific system versions and configurations used here.

In reference to our findings, several important questions arise, such as: “Does low-dose irradiation of the normal brain really matter?” and “What are its biological effects?” The widespread assumption is that lower levels of radiation delivered to normal brain tissue are clinically immaterial - unless one exceeds a somewhat arbitrarily defined V12 Gy threshold, which has now been shown to be associated with radiation-induced brain injury (RIBI). However, it should not escape the neuro-oncology community that the deleterious effects of “low-dose” WBRT on cognition went unrecognized for more than a generation, affecting many 10’s of millions of patients, largely because no one looked. While the anterior visual pathways are also known to have “unique” sensitivity to seemingly non-therapeutic doses of single fraction radiosurgery for malignant tumors, perhaps we are only aware of these clinical phenomena because their existence (blindness) is impossible to miss or deny? Maybe a similar “lower-dose” injury, i.e., alteration in normal brain function, happens routinely after SRS in more complex cognitive brain regions but goes clinically unrecognized because of our current failure to “look.” Recent large animal models inform us that radiation doses of less than 40 Gy will disrupt local neural circuits, which could be for better or worse. Meanwhile, doses ≥60 Gy are typically ablative, which is something we learned decades ago during the early days of radiosurgery [13]. Importantly, Fan et al. have recently shown in mice that radiation doses as low as 5 Gy can alter, seemingly permanently, brain circuitry (Journal article: Fan W, Maier J, Fu T, et al.: Single Shot Low-Dose Focal Radiation Durably Increases Cortical Excitability: A Potential Therapy for Neuronal Circuit Disorders. July 2025). It is reasonable to surmise that there is still much to be learned about the effects of low-dose radiation on the normal brain and the radiation thresholds for such effects.

In the present work, we largely focused on V5 Gy, V10 Gy, and V12 Gy dose levels to assess treatment planning quality without using the conformity index (CI) or the gradient index (GI). As more data on the effects of low-dose radiation emerge, it may well be that these long-used SRS quality parameters (CI and GI) are no longer adequate in assessing the safety profile of SRS platforms [5]. In fact, we question whether a new benchmark for assessing the quality of brain radiosurgery could be V5 Gy, or possibly even lower. Considering these emerging neuroradiobiology understandings, we urge researchers, when reporting physics and clinical results, to also start focusing on V5 Gy, V10 Gy, and V12 Gy dose levels, in addition to CI and GI.

## Conclusions

Herein, we demonstrate the superiority of dedicated cranial radiosurgery platforms in minimizing the irradiation of the uninvolved brain compared with multi-purpose linacs for the treatment of multiple small metastases. A significant range in the extent of brain volume being affected by lower doses was observed, with the largest being a 50-fold variation for V5 Gy. Although the level of radiation doses described in this analysis is generally low (V5 Gy, V10 Gy, and V12 Gy), emerging radiobiologic research suggests that even these small doses can profoundly and permanently alter normal brain circuitry. Therefore, we recommend that ensuing technical assessments of SRS platforms increasingly focus on the above measures as opposed to traditional parameters such as CI and GI. Finally, we encourage future clinical studies to carefully examine the extent to which lower-dose brain radiation may be unwittingly altering brain function and diminishing quality of life, especially in patients with multiple metastases.
